# Complications and length of stay after enhanced recovery after surgery compared to conventional care in colorectal cancer patients in Northern Italy

**DOI:** 10.3389/fsurg.2025.1694304

**Published:** 2025-12-11

**Authors:** Massimiliano Fabozzi, Federica Mereu, Francesco Marinelli, Isabella Bisceglia, Maurizio Zizzo, Andrea Morini, Fortunato Morabito, Magda Zanelli, Antonino Neri, Carmine Pinto, Lucia Mangone

**Affiliations:** 1 Surgical Oncology Unit, Azienda USL-IRCCS di Reggio Emilia, Reggio Emilia, Italy; 2 Epidemiology Unit, Azienda USL-IRCCS di Reggio Emilia, Reggio Emilia, Italy; 3 Gruppo Amici Dell'Ematologia Foundation-GrADE, Reggio Emilia, Italy; 4 Pathology Unit, Azienda USL-IRCCS di Reggio Emilia, Reggio Emilia, Italy; 5 Scientific Directorate, Azienda USL-IRCCS di Reggio Emilia, Reggio Emilia, Italy; 6 Oncology Unit, Azienda USL-IRCCS di Reggio Emilia, Reggio Emilia, Italy

**Keywords:** enhanced recovery, fast-track surgery, minimally invasive surgery, colorectal cancer, complications, length of hospital stay

## Abstract

**Background:**

This study aims to evaluate postoperative complications and length of stay in colorectal cancer patients undergoing ERAS vs. non-ERAS procedures in a population-based cohort in northern Italy.

**Methods:**

Patient characteristics (ASA, tumor site, stage, treatment, BMI) were used. Complications, stratified by Clavien-Dindo and length of stay (LOS), were reported. The odds ratio (OR) and 95% confidence interval (CI) were calculated to evaluate the difference between ERAS and non-ERAS patients.

**Results:**

A total of 319 patients were included, divided into the non-ERAS group (113 patients) and the ERAS group (206 patients). Non-ERAS vs. ERAS group showed more complications (16.8% vs. 13.6%; *p*-value 0.44) and more days of hospitalization (7.3 vs. 4.8; *p*-value < 0.01), but less re-surgery (3.5% vs. 4.4%; *p*-value 0.72), new hospitalizations (3.5% vs. 5.8%; *p*-value 0.37) and death at 30 days (0.9% vs. 1.5%; *p*-value 0.66). Multivariate analysis revealed an increased risk in patients with ASA 4 [OR 6.6; 95% CI 1.9–22.6] and a modest, non-significant increase risk in the non-ERAS group [OR 1.3; 95% CI 0.7–2.5].

**Conclusions:**

ERAS procedures appear to be able to allow intervention even in older patients and those with comorbidities, without compromising the results.

## Introduction

The Enhanced Recovery After Surgery (ERAS) protocol, initially introduced by Kehlet nearly 30 years ago in the pre-operative management of patients with colorectal cancer, has yielded significant benefits that have expanded over time (1, 2). Recent studies have confirmed that adherence to the ERAS (Enhanced Recovery After Surgery) protocol in colorectal cancer surgery is associated with a reduced length of stay (LOS), even among elderly patients (3, 4). This reduction is attributed to a lower incidence of postoperative complications (5, 6), including complications classified as Clavien-Dindo grade 3 or higher (4), which have also been observed in older patients (7).

While the correlation between postoperative complication reduction and enhanced 5-year survival is not unequivocal, studies do indicate a favorable impact on cancer-specific survival (8), accompanied by expeditious recovery of intestinal function (3, 9, 10) and a decline in inflammation indices (3, 9, 11).

Several studies have reported either a decrease in hospital readmissions (12, 13) or an increase in readmissions, albeit for minor pathologies (14). These improvements have the potential to result in enhanced patient satisfaction (15) and potential cost savings for healthcare systems (5).

This study aims to evaluate the incidence of postoperative complications and the duration of hospitalization in colorectal cancer patients who have undergone either ERAS or non-ERAS procedures in a population-based cohort study.

## Materials and methods

### The enhanced recovery after surgery protocols

The various ERAS Study Groups assembled in the early 2000s by Professor Fearon had and still have the aim of developing perioperative care and improving recovery through research, education, audit and implementation of evidence-based practices (16).

The protocols published by ERAS Society over the last 25 years have enabled a significant transformation in the perioperative management of any branch of surgery, delineating a more expeditious recovery trajectory (through effective pain management, mobilization, and early refeeding) and a reduction in postoperative complications (through the mitigation of risk factors and the maintenance of proper water and metabolic balance) (16–18).

### Study setting and inclusion criteria

Between 2021 and 2022, a Local Health Authority of Reggio Emilia multidisciplinary group was created to examine the applicability and potential short- and long-term benefits that adherence to the ERAS guidelines for elective colorectal surgery could bring to the management of colorectal cancer. Once the group's work was completed in February 2023, Surgical Oncology Unit adopted the 2018 ERAS Guidelines for Perioperative Care in Elective Colorectal Surgery (19) starting from March 1, 2023. Finally, an official Local Health Authority document was published authorizing the institutional implementation of the aforementioned guidelines starting from January 2024.

The study includes all adult patients (>18 years) who underwent elective colorectal surgery with a definitive histological examination proving colorectal cancer from March 2022 to September 2024 at the Surgical Oncology Unit of the Local Health Authority of Reggio Emilia—Scientific Institute for Research, Hospitalization and Health Care (IRCCS). Patients with American Society of Anesthesiologists (ASA) V, poor compliance, and undergoing emergency colorectal cancer surgery were excluded.

The list of patients was extracted from the Reggio Emilia Cancer Registry (CR), located in northern Italy, using specific search codes related to primary colorectal disease. The Registry has been in operation since 1996 and covers a population of 532,000 inhabitants. It is regarded as being of high quality due to the high rate of microscopic confirmation (93.2% for colorectal cancer) and the minimal percentage of Death Certificate Only (less than 0.1%) (20). The CR is responsible for collecting and analyzing data by established protocols, resulting in the production of statistics on various aspects of cancer, including incidence, mortality, prevalence, and survival rates. These statistics are disaggregated by demographic subgroups, as required by the epidemiological report outlined in Law No. 29 of March 22, 2019, which governs Cancer Registries in Italy. It is important to note that this legislation exempts the Registry from the obligation to obtain informed consent for data collection. The procedures for the epidemiological analysis of data collected by the Reggio Emilia CR have been approved by the Provincial Ethics Committee of Reggio Emilia (Protocol No. 2014/0019740, dated 4 August 2014).

### Data sources and data collection

The primary data sources utilized by the CR include pathological reports, hospital discharge records, and mortality data, supplemented with laboratory test results, diagnostic imaging reports, morbidity data (overall and major complications) obtained through retrospective review of the medical records, and information provided by general practitioners. Additionally, a case manager dedicated to the ERAS project collected data relating to patient compliance with individual items through outpatient meetings and/or telephone communications. Case identification was based on the International Classification of Diseases for Oncology, Third Edition (ICD-O-3), focusing on topography codes C18-C19 (21). A comprehensive data set was retrospectively retrieved from the hospital medical records, including information on the disease stage [according to the 8th edition of the TNM classification system (22)], surgical procedures, therapeutic interventions, and postoperative complications. Complications were categorized using the third edition of the Clavien-Dindo (C-D) classification system (23), which describes complications that can arise following surgical procedures. Finally, a multidisciplinary clinical audit was organized in 2025 to discuss the percentage results relating to the numerous outcomes of interest (including overall adherence and adherence to individual items of the ERAS protocol) obtained by the Accreditation and Quality Unit through the extraction of medical record data and case manager reports.

### Statistical methods

Patient characteristics, including gender, American Society of Anesthesiologists (ASA) Physical Status Classification System, tumor site (ascending colon, transverse colon, descending colon, rectum), stage, surgical approach (laparoscopy or laparotomy), chemotherapy and radiotherapy treatment, body mass index (BMI), age at diagnosis, were reported and stratified by the two study groups. Furthermore, postoperative complications requiring 30-day re-surgery, 30-day readmission, 30-day mortality, length of hospitalization, and the Clavien–Dindo postoperative morbidity classification were considered. The differences between the two groups were evaluated using Fisher's exact *χ*2 tests and the Wilcoxon rank-sum test for continuous variables. The odds ratio (OR) and the corresponding 95% confidence interval (CI) were calculated using logistic regression analysis to evaluate the effect of complications on the ERAS-non-ERAS procedure and other potential predictors, including age at diagnosis, American Society of Anesthesiologists (ASA) classification, and cancer stage. We also performed a linear regression to assess the association between LOS and ERAS, non-ERAS period, adjusting for the type of surgery. All analyses were performed using STATA 16.1 software (Stata Corp, College Station, TX, USA), and a *p*-value of < 0.05 was considered to indicate statistical significance.

### Ethics

This population-based cohort study utilized data from the Reggio Emilia Cancer Registry, which was approved by the Provincial Ethics Committee of Reggio Emilia (ref. no. 2014/0019740, dated 4 August 2014). Although individual consent was not obtained, the Ethics Committee authorized the use and processing of personal data, including information related to the health status of deceased or unreachable patients, for the purposes of this study.

## Results

The present study comprised 319 patients, who were divided into two groups: 113 patients undergoing conventional/non-ERAS management and 206 patients undergoing ERAS (Table 1). The ERAS group included a greater number of female patients, patients with ASA 3 comorbidities, descending colon site, stage III-IV at diagnosis, higher BMI and age at diagnosis, and underwent more laparoscopies (94.7% vs. 84.1%) and fewer laparotomies (5.3% vs. 15.9%; *p* value < 0.01). Overall adherence to the ERAS protocol was 72.7%.

**Table 1:** Reggio Emilia cancer registry 2022–2024. Characteristics of patients by gender, ASA, site, stage, treatment, BMI, and age at diagnosis in the non-ERAS and ERAS periods.

	All		non-ERAS group		ERAS group		*p*-value
	*N*	%	*n*	%	*n*	%	
Overall	319		113		206		
Gender							
Female	134	42.0	45	39.8	89	43.2	0.56
Male	185	58.0	68	60.2	117	56.8	
ASA							
1	16	5.0	8	7.1	8	3.9	0.26
2	152	47.8	55	48.7	97	47.3	
3	134	42.1	42	37.2	92	44.9	
4	16	5.0	8	7.1	8	3.9	
Site							
Ascending colon	139	43.6	51	45.1	88	42.7	0.88
Transverse colon	13	4.1	5	4.4	8	3.9	
Descending colon	73	22.9	23	20.4	50	24.3	
Rectum	94	29.5	34	30.1	60	29.1	
Stage							
I	80	25.1	29	25.7	51	24.8	0.74
II	89	27.9	35	31.0	54	26.2	
III	120	37.6	40	35.4	80	38.8	
IV	30	9.4	9	8.0	21	10.2	
Surgery							
Laparoscopy	290	90.9	95	84.1	195	94.7	<0.01
Laparotomy	29	9.1	18	15.9	11	5.3	
Neo chemotherapy							
Yes	53	16.6	15	13.3	38	18.5	0.23
No	266	83.4	98	86.7	168	81.5	
Neo radiotherapy							
Yes	50	15.7	18	15.9	32	15.5	0.93
No	269	84.3	95	84.1	174	84.5	
	mean	sd	mean	sd	mean	sd	
BMI	25.9	4.6	25.6	4.5	26.1	4.7	0.41
Age at diagnosis	70.6	12.9	70.0	13.3	70.9	12.7	0.63

BMI, body mass index; ASA, American society of anesthesiologists; SD, standard deviation.

**Figure 1:**
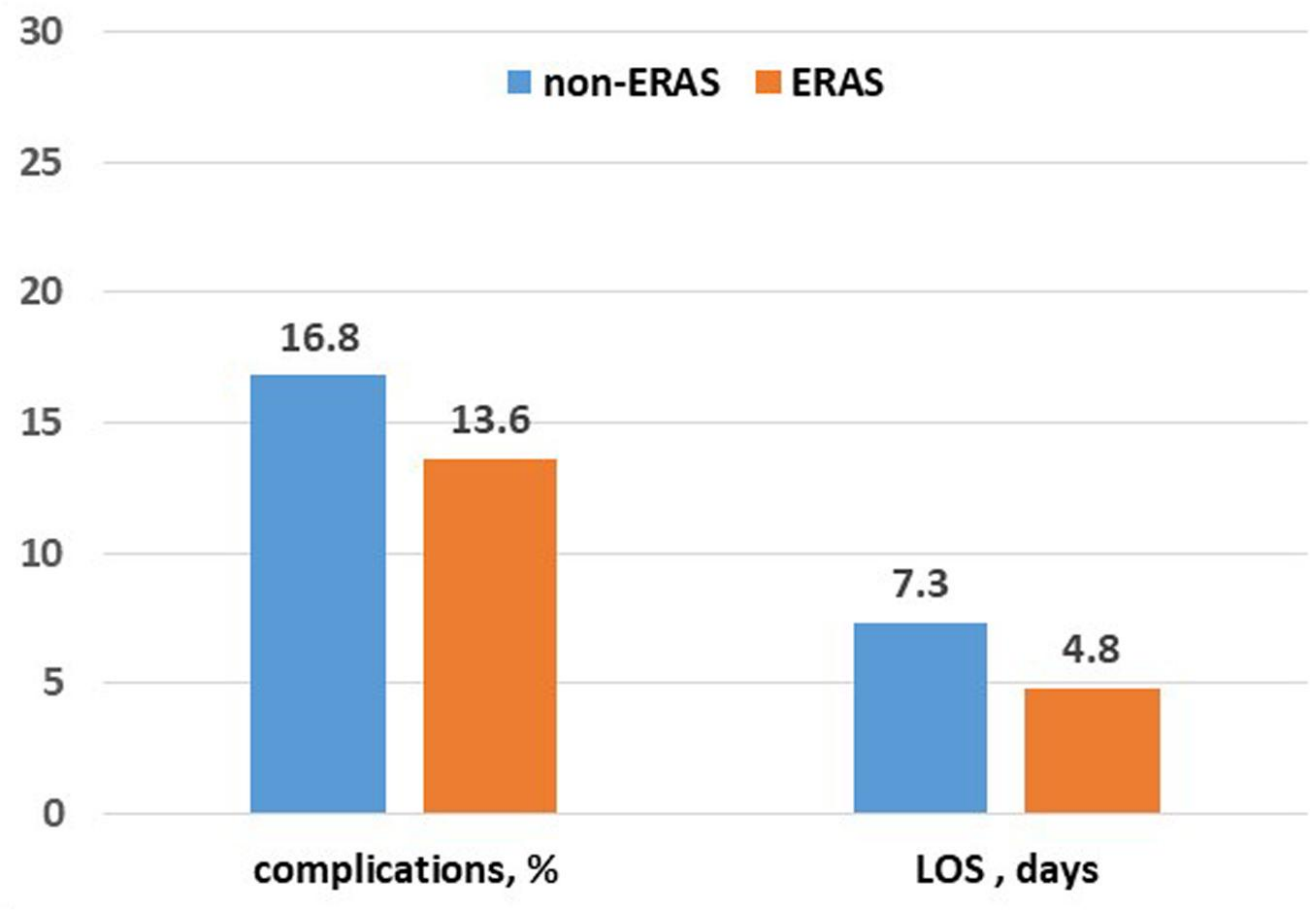
Reggio Emilia cancer registry 2022–2024. Percentage of complications and length of hospital stay in the ERAS and non-ERAS group.

**Figure 2:**
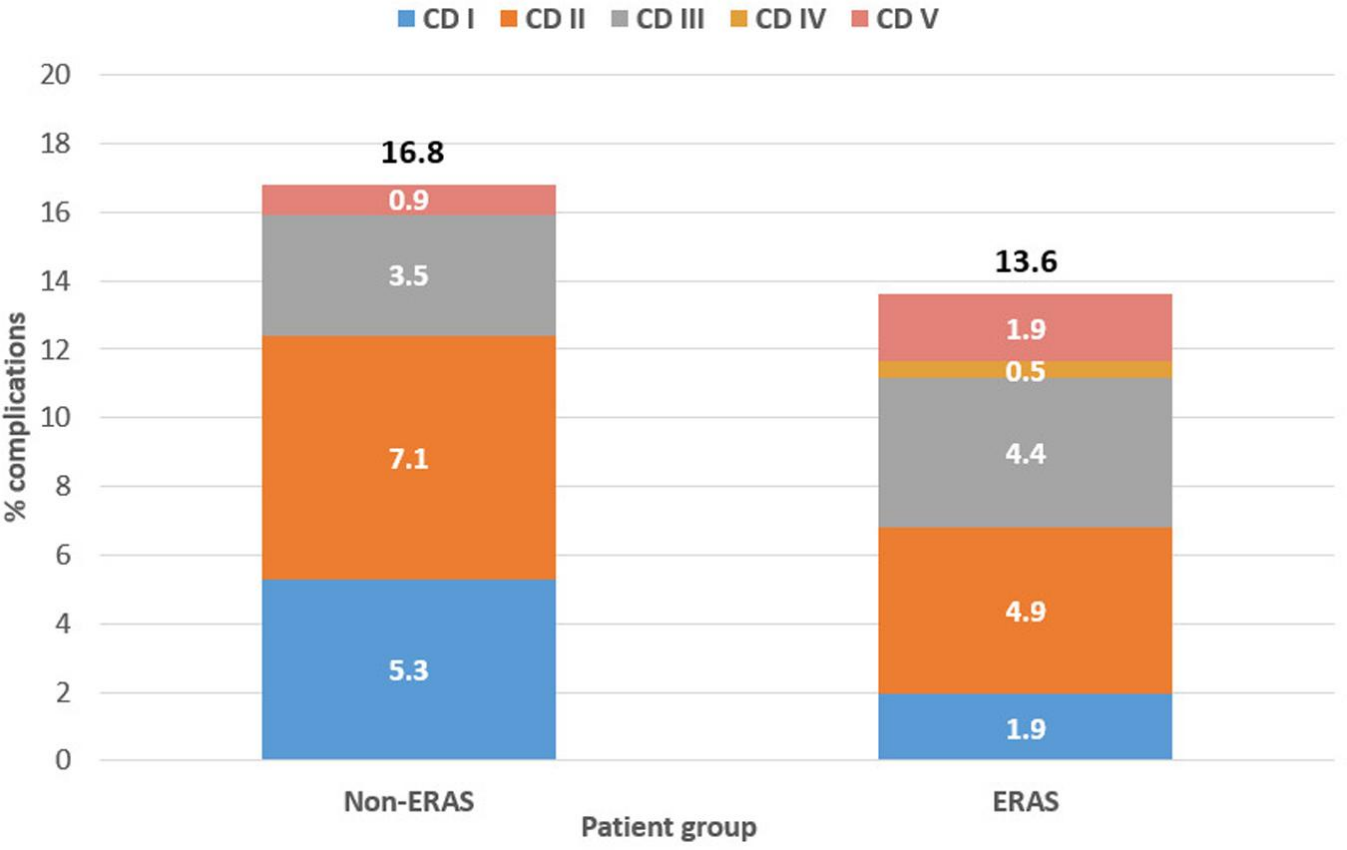
Reggio Emilia cancer registry 2022–2024. Distribution of complications, categorized by Clavien-Dindo, in the ERAS and non-ERAS group.

Postoperative complications were observed in 28 patients in the ERAS group and 19 patients in the non-ERAS group (13.6% and 16.8%, respectively; Table 2 and Figure 1). The ERAS group exhibited a higher incidence of re-surgery (4.4% vs. 3.5%), new recovery (5.8% vs. 3.5%), and deaths within 30 days (1.5% vs. 0.9%), although these differences were not statistically significant. However, the non-ERAS group demonstrated a significantly longer mean length of stay (7.3 days vs. 4.8 days; *p*-value < 0.01). Complications, categorized by Clavien-Dindo, are illustrated in Figure 2 and Table 3.

**Table 2:** Reggio Emilia cancer registry 2022–2024. Patient outcomes in terms of complications, reoperations, death, and days of hospitalization.

	non-ERAS group		ERAS group		*p*-value
	*N*	%	*n*	%	
Overall	113		206		
Complications					
Yes	19	16.8	28	13.6	0.44
No	94	83.2	177	86.4	
Clavien-Dindo (only for 47 complications)					
I	6	31.6	4	14.3	
II	8	42.1	10	35.7	
III	4	21.1	9	32.1	0.44
IV	0	0.0	1	3.6	
V	1	5.3	4	14.3	
Re-surgery at 30 days					
Yes	4	3.5	9	4.4	0.72
No	109	96.5	197	95.6	
New entry at 30 days					
Yes	4	3.5	12	5.8	0.37
No	109	96.5	194	94.2	
Death at 30 days					
Yes	1	0.9	3	1.5	0.66
No	112	99.1	128	98.5	
	Mean	sd	mean	sd	
Length of Stay (days)	7.3	4.5	4.8	3.8	<0.01

SD, standard deviation.

**Table 3:** Reggio Emilia cancer registry 2022–2024. Reasons for severe complications (Clavien-Dindo III-V) and re-surgery observed in the non-ERAS and the ERAS group.

	non ERAS group	ERAS group
Clavien-Dindo		
III	Intestinal obstruction due to an internal hernia	In a patient with cancer, the abdominal abscess was already present before surgery.
	Incarcerated hernia on trocar access	Anastomosis bleeding (endoscopic hemostasis)
	Dehiscence of the ileal stump of the ileo-colic anastomosis	Colonic perforation from supra-anastomotic colonic ischemia (patient with Clostridium difficile colitis)
	Intestinal obstruction without clear signs of internal hernia or other	Anastomosis bleeding (endoscopic hemostasis)
		Bleeding (re-operation)
		Intestinal obstruction from an internal hernia
		Rectovaginal fistula
		Intestinal obstruction from an internal hernia
		Ischemia anastomosis
IV		Intraoperative myocardial infarction
V	Death from postoperative liver failure in a cirrhotic patient	Intestinal obstruction (without evidence of mechanical causes), intestinal perforation, thalamic hemorrhage
		Postoperative neurological and vascular complications
		Medical complications and deterioration of general conditions in an already weakened patient
		Massive intestinal ischemia (mesenteric thrombosis already present)
Re-surgery		
	Exploratory laparotomy for intestinal obstruction without identifying mechanical causes	Closure of the stoma
	Re-operation for internal hernia	Hartmann procedure for supra-anastomotic ischemic perforation
	Hernia reduction on the trocar	Re-operation for intestinal perforation
	Exploratory laparoscopy and resection of the dehiscent ileal stump (stump of the ileo-colic anastomosis)	Re-operation for hemostasis in post-operative bleeding
		Re-operation for internal hernia
		Re-operation for internal hernia
		Operation for umbilical hernia
		Resection for anastomotic ischemia
		Re-operation for recto-vaginal fistula

Within the non-ERAS group, four C-D III complications were observed (two bowel obstructions, one hernia, and one dehiscence), and one C-D V complication due to liver failure in a patient with previous cirrhosis, which resulted in the patient's demise.

Within the ERAS group, nine C-D III complications were observed (three bleedings, two bowel obstructions, one abscess, one perforation, one fistula, one ischemia of the anastomosis), one C-D IV (intraoperative infarction in a patient with history of cardiovascular disease) and four C-D V (one occlusion with perforation that resulted in the patient's death 30 days after surgery, one neurological complication, one medical complication and one massive intestinal ischemia that led to the patient's death within 30 days of surgery).

The presence of comorbidities has been demonstrated to have a significant impact on the subsequent complications of patients (Table 4). Patients with ASA 3 and 4, who are more numerous in the enhanced recovery after surgery (ERAS) group, are more likely to develop severe complications with Clavien-Dindo ≥3.

**Table 4:** Reggio Emilia cancer registry 2022–2024. Type of complications, using the Clavien–Dindo classification, for ASA.

	ASA												Total
	non-ERAS group						ERAS group						
Clavien-Dindo	2		3		4		2		3		4		
I	2	33.3	3	33.3	1	25.0	3	30.0	1	7.1	0	0.0	10
II	2	33.3	4	44.4	2	50.0	3	30.0	6	42.9	1	25.0	18
III	2	33.3	2	22.2	0	0.0	4	40.0	4	28.6	1	25.0	13
IV	0	0.0	0	0.0	0	0.0	0	0.0	1	7.1	0	0.0	1
V	0	0.0	0	0.0	1	25.0	0	0.0	2	14.3	2	50.0	5
Total	6	100	9	100	4	100	10	100	14	100	4	100	47

ASA, American society of anesthesiologists.

Multivariate analysis reveals a marginal increase in the likelihood of complications, although not statistically significant, for males classified as ASA 3 and 4, as well as for those with stage II-IV. However, a substantial increase in risk is observed for males designated as ASA 4 (OR 6.6; 95% CI 1.9; 22.6). The non-ERAS group exhibits an elevated risk, although it does not reach statistical significance (OR 1.3; 95% CI 0.7; 2.5) (Table 5). Linear regression, adjusted for type of surgery, also shows that patients followed in the non-ERAS period had 2 more days of hospital stay (Coef: 2.02; 95% CI 1.11; 2.93) (data not shown).

**Table 5:** Logistic regression adjusted for ERAS status, age at diagnosis, gender, ASA, and stage.

	Logistic regression univariate analysis		Logistic regression multivariate analysis	
	OR	95% CI	OR	95% CI
Gender				
Female	1.0	Ref.	1.0	Ref.
Male	1.6	0.8–3.1	1.5	0.7–2.9
ASA				
2	1.0	Ref.	1.0	Ref.
3	1.8	0.9–3.5	1.5	0.7–3.3
4	8.5	2.8–25.8	6.6	1.9–22.6
Stage				
I	1.0	Ref.	1.0	Ref.
II	1.6	0.7–3.9	1.6	0.6–4.1
III	1.3	0.5–3.0	1.5	0.6–3.7
IV	1.3	0.3–4.4	1.8	0.5–6.6
Period				
ERAS	1.0	Ref.	1.0	Ref.
Non-ERAS	1.3	0.7–2.5	1.3	0.7–2.5

ASA, American society of anesthesiologists; OR, odds ratio; CI, confidence interval.

## Discussion

The ERAS protocol is a systematic approach to perioperative management that enhances surgical outcomes. Adherence to protocol guidelines is identified as a pivotal factor in achieving success (24, 25). A substantial body of research in the domain of elective colorectal surgery has demonstrated that the implementation of the ERAS protocol has been associated with several positive outcomes, including a reduction in hospital length of stay and morbidity (26, 27), as well as a decrease in postoperative complications and hospital readmissions (28). Nevertheless, adherence to ERAS remains challenging, with factors including patient-specific variables, socioeconomic status (SES), and institutional limitations (29–31).

In our setting, in March 2023, the ERAS protocol was implemented in patients with colorectal cancer, and the outcomes were evaluated in 206 patients who underwent ERAS and 113 patients who did not undergo ERAS. The ERAS group exhibited a tendency, albeit not statistically significant, to increase interventions in female patients with ASA 3 (more than double compared to the non-ERAS group), descending colon, advanced stages, older patients, and with higher BMI. The ERAS group demonstrated a substantial decline in laparotomy surgery (5.3% vs. 15.9%) in favor of laparoscopy (94.7% vs. 84.1%), coinciding with the implementation of the ERAS protocol. While ERAS procedures were administered to a greater number of patients with frailty and comorbidities, ERAS patients exhibited a lower incidence of complications (13.6% vs. 16.8%) and a reduced length of hospital stay (4.8 vs. 7.3 days).

Multivariate analysis confirmed a strong correlation between postoperative complications and the ASA score, as well as cancer stage, reinforcing the observation that the ERAS cohort consisted of higher-risk patients: non-ERAS patients exhibited a 30% higher, though not statistically significant, risk of complications, aligning with previous studies (5, 32). Despite the patients in the ERAS cohort of our study appearing to be slightly older and more fragile, this has had no impact on the number of interventions performed. Indeed, the number of laparoscopies has increased, thus enabling a greater number of patients to undergo this surgical approach. A review of the existing literature indicates that the benefits of ERAS are evident also in elderly patients, with a reduction in intensive care admissions (25.4% vs. 44.9%) (12) and a lower number of serious complications (4), thus reinforcing the idea that age alone should not exclude patients from ERAS protocols (7).

Current literature appears to show a significant reduction in overall postoperative complications following the adoption/implementation of ERAS, regardless of the surgical specialty considered (33–36). This impact, albeit less marked, also appears to extend to major postoperative complications (33–36). However, some relevant studies identified a trend towards a higher rate of major complications after colorectal surgery in the ERAS group (37). However, the authors seemed to consider the possibility that numerous biases could have influenced these results (37). Our results showed a higher rate of major postoperative complications in the ERAS group compared to the conventional one (6.79% vs. 4.42%). Several factors may have influenced the results obtained: learning curves of colorectal surgeons; complexity of the surgical procedures; extended resections. In the non-ERAS period, there were four colorectal surgeons, all of whom had good experience in laparoscopic colorectal surgery: two surgeons performed more than 35 cases per year and two more than 25 cases per year. In contrast, in the ERAS period, there were six surgeons performing colorectal surgery, two of whom had extensive experience (more than 35 cases per year) and four who were still learning (fewer than 15 cases per year). Furthermore, in the non-ERAS period the laparoscopic procedures performed were exclusively the standardized (D2 lymphadenectomy) right hemicolectomy, left hemicolectomy, sigmoidectomy and anterior rectal resection. Instead, during the ERAS period several other complex laparoscopic procedures were performed: transverse colectomy with intracorporeal or extracorporeal anastomosis, resection of the splenic flexure with intracorporeal or extracorporeal anastomosis, extended left colectomy with intracorporeal anastomosis, ultralow anterior rectal resection with coloanal anastomosis, and D3 lymphadenectomies. Finally, colorectal resections performed during the ERAS period included a higher number of multivisceral procedures than those performed in the non-ERAS period.

The ERAS group has been shown to exhibit a reduced number of hospitalization days, as documented in the existing literature. The data consistently demonstrate a decline in the number of hospitalizations observed among ERAS patients. The findings of the present study, in conjunction with those of previous research (5, 6, 32), suggest a correlation between a reduced recovery period and various treatment durations. The specific durations in question are 9 vs. 14 days (6), 9 vs. 12 days (5), and 7.5 vs. 8.5 days (32). The present study also builds upon the results of a previous investigation (14), which reported a recovery period of 5 days vs. 8.1 days. The findings of this study suggest that the enhanced recovery period is attributable to the implementation of perioperative care strategies, including early mobilization, optimal pain management, and early nutrition.

However, there was an increase in 30-day readmissions (5.8% vs. 3.5%) and re-surgeries (4.4% vs. 3.5%) in ERAS patients. This finding is of significance, as the increased number of readmissions in the ERAS group, particularly those related to sub-occlusive syndrome, pre-existing abdominal abscesses, and surgical complications, can be attributed to the greater complexity of patients treated under the ERAS protocol, including those with advanced disease or higher ASA scores.

A total of nine ERAS patients required re-surgical procedures within 30 days. While this may raise concerns about the efficacy of the protocol, the nature of the re-operations, including those necessitated by postoperative hemorrhage and intestinal obstruction, serves to underscore the intricate nature of ERAS patients. A significant proportion of these patients had undergone multivisceral resections or were afflicted with pre-existing conditions.

Four postoperative deaths occurred: three in the ERAS group and one in the non-ERAS group. All fatalities were associated with pre-existing conditions. The patient who died in the non-ERAS group had cirrhosis. Among the three deaths in the ERAS group, one had pre-existing mesenteric thrombosis, one was already in a markedly debilitated condition, and one had a history of neurological and cardiovascular complications.

This finding reinforces the notion that patients with greater complexity and comorbidity are being included, necessitating a more comprehensive approach to patient management. This emphasizes the significance of personalized patient management within the ERAS framework, where conservative management can be prioritized in cases where surgery is not immediately necessary. A survey of the literature reveals comparable trends, with similar rates of minor surgical interventions observed in the ERAS and non-ERAS cohorts (80% vs. 77%) (5). Furthermore, in some instances, lower 30-day readmission rates have been documented in ERAS patients (6.6% vs. 8.3%) (13). A recent French study highlights additional independent risk factors in ERAS patients, including age, BMI, smoking, ASA >3, and laparotomy (38), reinforcing the importance of tailored perioperative strategies.

It is evident that adherence to ERAS protocols has a substantial impact on patient outcomes (6, 39–41), with mounting evidence underscoring the significance of inflammatory marker control (IL-6, CRP) (3, 9), optimized pain management and nutrition (42), and early restoration of bowel function (43). Multidisciplinary approaches to ERAS implementation (10), as previously reported in our institutional studies (44), have the potential to further enhance outcomes, particularly in high-risk populations (45). Moreover, ERAS adoption has been demonstrated to be associated with potential cost reductions due to decreased hospitalization times and streamlined postoperative care (5).

At our center, the only patients considered ineligible for ERAS protocol are those classified as ASA V or those who explicitly stated in advance their unwillingness to follow the protocol's recommendations. Since the implementation of the ERAS protocol in March 2023, no ASA V patients have been admitted, and no patient has declined to adhere to the protocol's procedures.

In the present cohort, patients with stage I colorectal cancer were referred to the relevant gastroenterology department for follow-up. In contrast, those with stage II-IV disease were managed by the medical oncology department for adjuvant or palliative treatment, according to institutional guidelines. It is imperative to acknowledge the role of SES factors in the context of ERAS implementation, as disparities have the potential to influence compliance and postoperative outcomes (46). Modifications and refinements to ERAS protocols may further mitigate postoperative complications and enhance recovery (47). Finally, patient-reported satisfaction, as highlighted in the literature (15), is an essential factor that should be integrated into future evaluations.

A significant strength of this study is the incorporation of real-world, population-based data up to 2024, thereby minimizing selection bias. A key limitation is the emphasis on short-term outcomes, with a paucity of long-term oncological follow-up, particularly regarding the impact of ERAS-related complications on subsequent cancer treatments.

## Conclusions

Colorectal cancer continues to be a significant public health problem, characterized by high incidence and mortality rates. Although screening programs continue to detect tumors at early stages, optimizing surgical management is crucial to improving patient outcomes. The results of this study suggest that, despite some limitations, the ERAS procedure appears to facilitate effective perioperative care for colorectal cancer, thus enabling the treatment of increasingly complex patients over time.

The reduction in surgical complications (e.g., anastomotic dehiscence) is not always significant, whereas a more consistent decrease is observed in medical or non-surgical complications (48). The occurrence of severe events within the ERAS group likely reflects the presence of high-risk factors and the inclusion of complex patients undergoing demanding surgical procedures.

## Data Availability

The raw data supporting the conclusions of this article will be made available by the authors, without undue reservation.

## References

[1] Kehlet H. Multimodal approach to control postoperative pathophysiology and rehabilitation. Br J Anaesth. (1997) 78:606–17. 10.1093/bja/78.5.606 9175983

[2] Kehlet H Mogensen T. Hospital stay of 2 days after open sigmoidectomy with a multimodal rehabilitation programme. Br J Surg. (1999) 86:227–30. 10.1046/j.1365-2168.1999.01023.x 10100792

[3] Han H Wan R Chen J Fan X Zhang L. Effects of the enhanced recovery after surgery (ERAS) protocol on the postoperative stress state and short-term complications in elderly patients with colorectal cancer. Cancer Rep (Hoboken). (2024) 7(2):e1979. 10.1002/cnr2.1979 38351544 PMC10864734

[4] Navarra A Porcellini I Mongelli F Popeskou SG Grass F Christoforidis D. Long-term outcomes in elderly patients after elective surgery for colorectal cancer within an ERAS protocol: a retrospective analysis. Langenbecks Arch Surg. (2023) 408(1):438. 10.1007/s00423-023-03179-7 37978074

[5] Ni X Jia D Chen Y Wang L Suo J. Is the enhanced recovery after surgery (ERAS) program effective and safe in laparoscopic colorectal cancer surgery? A meta-analysis of randomized controlled trials. J Gastrointest Surg. (2019) 23(7):1502–12. 10.1007/s11605-019-04170-8 30859422

[6] Sato H Ota H Munakata K Matsuura Y Fujii M Wada N Perioperative fluid management influences complication rates and length of hospital stay in the enhanced recovery after surgery (ERAS) protocol for patients with colorectal cancer. Surg Today. (2023) 53(2):242–51. 10.1007/s00595-022-02568-7 35933631

[7] Pędziwiatr M Pisarska M Wierdak M Major P Rubinkiewicz M Kisielewski M The use of the enhanced recovery after surgery (ERAS) protocol in patients undergoing laparoscopic surgery for colorectal cancer–A comparative analysis of patients aged above 80 and below 55. Pol Przegl Chir. (2015) 87(11):565–72. 10.1515/pjs-2016-0004 26816404

[8] Crippa J Calini G Santambrogio G Sassun R Siracusa C Maggioni D ERAS protocol applied to oncological colorectal mini-invasive surgery reduces the surgical stress response and improves long-term cancer-specific survival. Surg Laparosc Endosc Percutan Tech. (2023) 33(3):297–301. 10.1097/SLE.0000000000001181 37184246

[9] Zhang Q Sun Q Li J Fu X Wu Y Zhang J The impact of ERAS and multidisciplinary teams on perioperative management in colorectal cancer. Pain Ther. (2025) 14(1):201–15. 10.1007/s40122-024-00667-6 39499490 PMC11751192

[10] Kannan V Ullah N Geddada S Ibrahiam A Munaf Shakir Al-Qassab Z Ahmed O Impact of “enhanced recovery after surgery” (ERAS) protocols vs. traditional perioperative care on patient outcomes after colorectal surgery: a systematic review. Patient Saf Surg. (2025) 19(1):4. 10.1186/s13037-024-00425-9 39819478 PMC11737126

[11] Alves Bersot CD Ferreira Gomes Pereira L Goncho VGV Pereira JEG Falcão LFDR. Enhancing recovery and reducing inflammation: the impact of enhanced recovery after surgery recommendations on inflammatory markers in laparoscopic surgery—a scoping review. Front Surg. (2024) 11:1450434. 10.3389/fsurg.2024.1450434 39717352 PMC11663872

[12] Martínez-Escribano C Arteaga Moreno F Cuesta Peredo D Blanco Gonzalez FJ De la Cámara-de Las Heras JM Tarazona Santabalbina FJ. Before-and-After study of the first four years of the enhanced recovery after surgery (ERAS®) programme in older adults undergoing elective colorectal cancer surgery. Int J Environ Res Public Health. (2022) 19(22):15299. 10.3390/ijerph192215299 36430017 PMC9691222

[13] Shida D Tagawa K Inada K Nasu K Seyama Y Maeshiro T Enhanced recovery after surgery (ERAS) protocols for colorectal cancer in Japan. BMC Surg. (2015) 15:90. 10.1186/s12893-015-0079-0 26215107 PMC4517644

[14] Mangone L Mereu F Zizzo M Morini A Zanelli M Marinelli F Outcomes before and after implementation of the ERAS (enhanced recovery after surgery) protocol in open and laparoscopic colorectal surgery: a comparative real-world study from Northern Italy. Curr Oncol. (2024) 31(6):2907–17. 10.3390/curroncol31060222 38920706 PMC11202664

[15] Zou M Xu J Chen F Wang N Long S Wu H A qualitative exploration of perioperative subjective experiences of colorectal cancer patients undergoing fast-track surgery. Sci Rep. (2024) 14(1):30721. 10.1038/s41598-024-79944-5 39730444 PMC11681125

[16] History—ERAS Society. ERAS. Available online at: https://erassociety.org/about/history/ (Accessed October 25, 2025).

[17] Ripollés-Melchor J Abad-Motos A Zorrilla-Vaca A. Enhanced recovery after surgery (ERAS) in surgical oncology. Curr Oncol Rep. (2022) 24(9):1177–87. 10.1007/s11912-022-01282-4 35403970

[18] Mithany RH Daniel N Shahid MH Aslam S Abdelmaseeh M Gerges F Revolutionizing surgical care: the power of enhanced recovery after surgery (ERAS). Cureus. (2023) 15(11):e48795. 10.7759/cureus.48795 38024087 PMC10646429

[19] Gustafsson UO Scott MJ Hubner M Nygren J Demartines N Francis N Guidelines for perioperative care in elective colorectal surgery: enhanced recovery after surgery (ERAS®) society recommendations: 2018. World J Surg. (2019) 43(3):659–95. 10.1007/s00268-018-4844-y 30426190

[20] Mangone L Borciani E Michiara M Vicentini M Carrozzi G Mancuso P I Tumori Nelle Province Dell’Area Vasta Emilia Nord: Piacenza, Parma, Reggio Emilia e Modena: Anni 2013–2014. Modena, Italy: Associazione Italiana Registri Tumori (2015).

[21] Fritz A Percy C Jack A Shanmugaratnam K Sobin L Parkin DM International Classification of Diseases for Oncology. 3rd ed. Geneva, Switzerland: World Health Organization (2013).

[22] Sobin L Gospodarowicz M Wittekind C. TNM Classification of Malignant Tumours. 8th ed. Geneva, Switzerland: UICC (2011). Raffaello Cortina Editore: Milan, Italy.

[23] Dindo D Demartines N Clavien PA. Classification of surgical complications: a new proposal with evaluation in a cohort of 6336 patients and results of a survey. Ann. Surg. (2004) 240:205–13. 10.1097/01.sla.0000133083.54934.ae 15273542 PMC1360123

[24] Gustafsson UO Hausel J Thorell A Ljungqvist O Soop M Nygren J. Adherence to the enhanced recovery after surgery protocol and outcomes after colorectal cancer surgery. Arch Surg. (2011) 146(5):571–7. 10.1001/archsurg.2010.309 21242424

[25] Berian JR Ban KA Liu JB Ko CY Feldman LS Thacker JK. Adherence to enhanced recovery protocols in NSQIP and association with colectomy outcomes. Ann Surg. (2019) 269(3):486–93. 10.1097/SLA.0000000000002566 29064887

[26] McAtee EE Talukder AM Davenport DL Bhakta AS Patel JA. A review of quality improvement process measures for colorectal surgery in the United States using the American college of surgeons national surgical quality improvement program database - have we made progress? Am Surg. (2023) 89(6):2976–8. 10.1177/00031348221101523 35537489

[27] Fields AC Dionigi B Scully RE Stopfkuchen-Evans MF Maldonado L Henry A Reduction in cardiac arrhythmias within an enhanced recovery after surgery program in colorectal surgery. J Gastrointest Surg. (2020) 24(5):1158–64. 10.1007/s11605-019-04298-7 31228081

[28] Antoniv M Nikiforchin A Sell NM Bordeianou LG Francone TD Ahmed F Impact of multi-institutional enhanced recovery after surgery protocol implementation on elective colorectal surgery outcomes. J Am Coll Surg. (2025) 240(2):158–66. 10.1097/XCS.0000000000001202 39812414

[29] Smith BP Jones BA Cofer KD Hollis RH Shao C Gleason L Racial disparities in postoperative outcomes persist for patients with inflammatory bowel disease under a colorectal enhanced recovery program. Am J Surg. (2023) 226(2):227–32. 10.1016/j.amjsurg.2023.04.009 37120415 PMC10524897

[30] Wahl TS Goss LE Morris MS Gullick AA Richman JS Kennedy GD Enhanced recovery after surgery (ERAS) eliminates racial disparities in postoperative length of stay after colorectal surgery. Ann Surg. (2018) 268(6):1026–35. 10.1097/SLA.0000000000002307 28594746 PMC9945468

[31] Leeds IL Alimi Y Hobson DR Efron JE Wick EC Haut ER Racial and socioeconomic differences manifest in process measure adherence for enhanced recovery after surgery pathway. Dis Colon Rectum. (2017) 60(10):1092–101. 10.1097/DCR.0000000000000879 28891854 PMC5647878

[32] Pagano E Pellegrino L Rinaldi F Palazzo V Donati D Meineri M Implementation of the ERAS (enhanced recovery after surgery) protocol for colorectal cancer surgery in the piemonte region with an audit and feedback approach: study protocol for a stepped wedge cluster randomised trial: a study of the EASY-NET project. BMJ Open. (2021) 11(6):e047491. 10.1136/bmjopen-2020-047491 34083345 PMC8183289

[33] Feng JY Wang SF Yan J. The application of enhanced recovery after surgery for gastrectomy and colorectal resection: a systematic review and meta-analysis. J Laparoendosc Adv Surg Tech A. (2023) 33(6):586–95. 10.1089/lap.2023.0036 37130316

[34] Li N Liu Y Chen H Sun Y. Efficacy and safety of enhanced recovery after surgery pathway in minimally invasive colorectal cancer surgery: a systemic review and meta-analysis. J Laparoendosc Adv Surg Tech A. (2023) 33(2):177–87. 10.1089/lap.2022.0349 36074099

[35] Slim N Teng WH Shakweh E Sylvester HC Awad M Schembri R Enhanced recovery programme after colorectal surgery in high-income and low-middle income countries: a systematic review and meta-analysis. Int J Surg. (2023) 109(11):3609–16. 10.1097/JS9.0000000000000644 37598350 PMC10651249

[36] Sauro KM Smith C Ibadin S Thomas A Ganshorn H Bakunda L Enhanced recovery after surgery guidelines and hospital length of stay, readmission, complications, and mortality: a meta-analysis of randomized clinical trials. JAMA Netw Open. (2024) 7(6):e2417310. Erratum in: JAMA Netw Open. 2024 July 1;7(7):e2428433. doi: 10.1001/jamanetworkopen.2024.28433. 10.1001/jamanetworkopen.2024.17310 38888922 PMC11195621

[37] Ripollés-Melchor J Abad-Motos A Cecconi M Pearse R Jaber S Slim K Association between use of enhanced recovery after surgery protocols and postoperative complications in colorectal surgery in Europe: the EuroPOWER international observational study. J Clin Anesth. (2022) 80:110752. 10.1016/j.jclinane.2022.110752 35405517

[38] Tidadini F Trilling B Sage PY Durin D Foote A Quesada JL Five-year oncological outcomes after enhanced recovery after surgery (ERAS) compared to conventional care for colorectal cancer: a retrospective cohort of 981 patients. Tech Coloproctol. (2024) 29(1):9. 10.1007/s10151-024-03036-9 39641815

[39] Milone M Elmore U Manigrasso M Ortenzi M Botteri E Arezzo A ERas and COLorectal endoscopic surgery: an Italian society for endoscopic surgery and new technologies (SICE) national report. Surg Endosc. (2022) 36(10):7619–27. Erratum in: Surg Endosc. 2022 Oct;36(10):7628. doi: 10.1007/s00464-022-09344-1. 10.1007/s00464-022-09212-y 35501602 PMC9485180

[40] Pal AR Mitra S Aich S Goswami J. Existing practice of perioperative management of colorectal surgeries in a regional cancer institute and compliance with ERAS guidelines. Indian J Anaesth. (2019) 63(1):26–30. 10.4103/ija.IJA_382_18 30745609 PMC6341895

[41] Anifalaje OK Ojo C Balogun OT Ayodele FA Azeez A Gabriels S. The determinants of long-term outcomes after colorectal cancer surgery: a literature review. Cureus. (2024) 16(12):e74985. 10.7759/cureus.74985 39744296 PMC11692687

[42] Yeom J Lim HS. Effects of the enhanced recovery after surgery (ERAS) program for colorectal cancer patients undergoing laparoscopic surgery. Clin Nutr Res. (2022) 11(2):75–83. 10.7762/cnr.2022.11.2.75 35558997 PMC9065394

[43] Zargar T Wagay BA Banday I Haq MF Parray FQ Banday M The effect of chewing gum on the return of bowel activity after colorectal cancer surgery. Euroasian J Hepatogastroenterol. (2024) 14(2):210–3. 10.5005/jp-journals-10018-1456 39802852 PMC11714110

[44] Mangone L Zizzo M Nardecchia M Marinelli F Bisceglia I Braghiroli MB Impact of multidisciplinary team management on survival and recurrence in stage I-III colorectal cancer: a population-based study in Northern Italy. Biology (Basel). (2024) 13(11):928. 10.3390/biology13110928 39596883 PMC11592292

[45] Albalawi HIH Alyoubi RKA Alsuhaymi NMM Aldossary FAK Mohammed GAA Albishi FM Beyond the operating room: a narrative review of enhanced recovery strategies in colorectal surgery. Cureus. (2024) 16(12):e76123. 10.7759/cureus.76123 39840197 PMC11745840

[46] Antoniv M Nikiforchin A Sell NM Bordeianou LG Francone TD Ahmed F Impact of multi-institutional enhanced recovery after surgery protocol implementation on elective colorectal surgery outcomes. J Am Coll Surg. (2025) 240(2):158–66. 10.1097/XCS.0000000000001202 39812414

[47] Wong CS Zaman S Siddiraju K Sellvaraj A Ghattas T Tryliskyy Y. Effects of enteral immunonutrition in laparoscopic versus open resections in colorectal cancer surgery: a meta-analysis of randomised controlled trials. Eur J Surg Oncol. (2024) 51(2):109488. 10.1016/j.ejso.2024.109488 39708458

[48] Greco M Capretti G Beretta L Gemma M Pecorelli N Braga M. Enhanced recovery program in colorectal surgery: a meta-analysis of randomized controlled trials. World J Surg. (2014) 38(6):1531–41. 10.1007/s00268-013-2416-8 24368573

